# Analysis of central and peripheral PTH decay values in patients with primary hyperparathyroidism^☆^^☆☆^

**DOI:** 10.1016/j.bjorl.2025.101606

**Published:** 2025-05-22

**Authors:** Giovanna Luiza Caxeiro, Davi Knoll Ribeiro, Rafael Dias Romero, Marcello Rosano, Murilo Catafesta Neves, Marcio Abrahao

**Affiliations:** Universidade Federal de São Paulo (UNIFESP), São Paulo, SP, Brazil

**Keywords:** Parathyroidectomy, Intraoperative parathyroid hormone, Primary hyperparathyroidism

## Abstract

**Objective:**

The aim of this study was to analyze the peculiarities of the collection sites.

**Methods:**

It is a prospective study of patients undergoing parathyroidectomy by HPTP, with PTH values in the peripheral collection and from the ipsilateral VJI at times: initial (T0) and 10-min after removal of the diseased gland (T10).

**Results:**

61 participants were evaluated. The median PTH at baseline was 147.9 in the peripheral vein and 476.58 in the central vein. The median at T10 was 36 in the peripheral vein and 33 in the central vein. The central vein values showed a greater reduction, with the peripheral values showing a median decay of 74.35%, while the central values dropped by 82.38%. In the initial collection, PTH values were higher in the central vein, while they tended towards homeostasis after the removal of the diseased gland, regardless of the collection site. Thus, the average decay values from the central vein collections were higher and more significant than those from the peripheral veins.

**Conclusion:**

The use of central values thus implies greater reliability for to intraoperative monitoring, with long-term comparative studies still being necessary to determine more targeted and effective surgical approaches.

**Level of Evidence:**

Level III.

## Highlights

• The minimally invasive parathyroidectomy is a procedure in which only the diseased parathyroid gland is removed.
• PTH measurement collected from central jugular vein may allows the surgeon to quantitatively determine the excision of all hyperfunctioning parathyroid tissue more precisely.
• Collections from peripheral and central sites were found to be effective in this study.
• There is a tendency to use values collected from central sites, as they confirmed the ideal reduction in values following removal.

## Introduction

Primary Hyperparathyroidism (PHPT) is an endocrine disease caused by a primary disease of the parathyroid glands, which leads to their hyperfunctioning. It is characterized by hypercalcemia and increased or abnormal Parathyroid Hormone (PTH) values. Parathyroidectomy (PTx) is the only curative approach for this disease.^1^ Initially, Bilateral Neck Exploration (BNE) was used in patients with PHPT with resection of enlarged parathyroid glands and without any preoperative localization studies, resulting in a success rate greater than 95%.^6^

Preoperative localization exams and new surgical techniques are increasingly studied in order to provide a safer, more effective surgical approach with less morbidity. One of the methods studied is Minimally Invasive Parathyroidectomy (MIP), in which only the diseased gland is removed, without bilateral exploration of the neck. Advantages include a lower incidence of hypocalcemia, similarly high cure rates with lower complication rates, shorter hospital stay and lower hospital costs.^2^, ^6^

An important component of MIP is the intraoperative PTH assay, which was established in 1988. Improvements to this method are being investigated, which could provide important value, for example, for patients with negative preoperative localization imaging, and it is possible to use differential jugular venous sampling to lateralize the side of the neck with the hyperfunctioning parathyroid gland.^6^

Intraoperative PTH measurement (IO-PTH) allows the surgeon to quantitatively determine the excision of all hyperfunctioning parathyroid tissue, predicting long-term operative success with less morbidity.^3^ The PTH sample can be collected from a Peripheral or Central Vein (Internal Jugular Vein). A greater than 50% reduction in PTH levels after 10 min, from the starting value is considered a biological cure.^2^, ^3^ Each collection site presents certain advantages. Our study was planned in order to highlight the differences in the distinct use for intraoperative PTH collection from Peripheral and Central Veins, analyzing the tendencies of values according to the collection location and their benefits for the best surgical approach.

## Methods

This is a prospective study, from January 2018 to January 2021, of all patients undergoing PHPT parathyroidectomy at a tertiary hospital by the Head and Neck Surgery team. Data about sex, age, location, exams, and surgeries performed were collected from medical records.

As a study protocol, after cervical incision and dissection of the prethyroid muscles to the bilateral carotid sheath, the internal jugular veins were identified, and PTH was collected under direct view of the internal jugular vein, at its lowest point. After PTH collection, exploration and identification of the four parathyroid glands was performed. A second PTH collection was performed from the ipsilateral jugular vein to the diseased parathyroid gland 10-min after the removal of the diseased parathyroid.

Inclusion criteria were patients diagnosed with PPH and eligible for surgery who had PTH values in the peripheral collection and in the ipsilateral jugular vein to the disease at the times: beginning of surgery and 10-min after. Exclusion criteria were patients with suspected or diagnosed multiglandular disease, in addition to patients under 18-years of age.

Statistical analysis for non-parametric data using the Wilcoxon test was performed, presenting data in quartiles and medians.

## Results

Our study included a total of 61 participants, mostly female (78.6%), with 13 men and 48 women. The average age was 55 years.

The median PTH at baseline (T0) was 147.9 in the Peripheral site (PV) collection, with a quartile of 25 of 89 and a quartile of 75 of 187, while it was 476.58 in the Central site Collection (CV), with a quartile of 25 out of 144 and a quartile of 75 out of 568. A comparison of the two values shows a statistical difference (*p* = 0.00002774) (Table 1).

**Table 1:** Comparison of PTH values from central and peripheral collection measured 10-min after removal of the pathological parathyroid gland and in relation to decay.

	Central PTH	Peripheral PTH
PTH T10	33	36
PTH decay	82.38%	74.35%

The median obtained for ten minutes after removal of the diseased gland (T10) was 36 in peripheral values, with a quartile of 25 of 26 and a quartile of 75 of 33, and in the central values of 33, with the quartile of 25 out of 28 and the quartile of 75 out of 52. Notably, although the mean of the IO-PTH values at T0 were very diverse, at T10 the IO-PTH values presented statistically similar results regardless of the collection site (*p* = 0.8137) (Table 2).

**Table 2:** Comparison of median PTH values collected at the beginning of surgery and 10-min after removal of the diseased parathyroid gland in peripheral and central sites.

	T0	T10
Central PTH	476.58	33
Peripheral PTH	147.9	36

It was possible to observe that the central values presented a greater decrease than those obtained from the peripheral collections, and the peripheral values presented a median decay of 74.35%, with the 25 quartiles of 61.73 and the quartile of 75 of 81.04%, while the central values, 82.38%, with the 25th quartile of 75% and the 75th quartile of 91.14%. These values are statistically different (*p* = 0.00006087).

## Discussion

The study showed that, as previously indicated in the literature, both IO-PTH values collected from Peripheral (PV), and Central (CV) sites are valid for intraoperative monitoring of PTH reductions. Neves et al. had already concluded that the IO-PTH shows reliable results, with a cure correlated with a drop in values and disease persistence when they remain stable. Thus, IO-PTH measurement proved to be effective in predicting surgical success in primary hyperparathyroidism.^5^

Corroborating what was indicated by Ito et al. central IO-PTH values show a more notable decrease when compared to peripheral values, being more accurate in determining the hyperfunctioning gland and able to contribute to a more targeted surgery.^5^ We also noted in our study that although higher PTH levels are measured from central venous samples, the accuracy of IO-PTH dynamics is generally not affected by sample location.^2^, ^3^

According to Woodrum et al., when comparing the PV and VT values, both proved to be useful to carry out intraoperative monitoring of PTH reductions. Considering that baseline levels are higher in CV, a trend was observed in the 2004 study in which the CV group was less likely to have decreased to the normal range in 10-min, requiring a 15-min period to reach a reduction. In our study, analyzing the 10-min period, a 50% reduction from the initial values for the central site was observed, proving its efficiency for MIP.

An important point highlighted in this study was the homeostasis of the PTH results after the removal of the diseased gland since they were shown to be statistically equivalent regardless of the collection site. This information is relevant for more accurate monitoring of IO-PTH, in line with the physiological principle used for the analysis of PTH reduction after withdrawal.^7^, ^8^ The functioning of healthy glands is diminished due to the negative feedback mechanism stemming from increased serum calcium typical of PPHTH. The main regulatory factor for PTH secretion is the concentration of ionic calcium in the extracellular fluid.^11^ When removing the hyperfunctioning gland, which was responsible for maintaining the PTH levels elevated, it is expected that they will decrease, as the response of healthy parathyroids is not immediate. The concept of intraoperative monitoring of PTH is effective due to knowledge about the short half-life of this hormone (approximately 3−4 min).^4^ Our study showed that the range of PTH values when removing the hyperfunctioning gland is similar in all regions of the body.

In order to monitor intraoperative PTH, several protocols were created for the use of ideal parameters for reductions in PTH levels to determine surgical outcomes. Foley et al. evaluated the most frequently used methods in their study and found that ideal PTH decay values are within the range of 69%–72%, as they have greater specificity and sensitivity.^9^, ^10^ In our current research, the mean decay values observed both from the peripheral and central collections were within the range determined by Foley. However, it was observed that the decay of CV (82.38%) is higher than that for PV (74.35%). It is possible to infer that the use of central values would correspond to greater effectiveness of intraoperative monitoring of PTH values, since higher ranges and those above the level considered ideal in previous studies indicate a higher success rate in targeted surgeries and a lower frequency of approaches in unnecessary glands.

Future investigations need to use a larger sample to observe reduction patterns in parathyroid hormone in central or peripheral sites, regardless of similar final values, after the removal of the diseased gland, in order to confirm the greater sensitivity and specificity in the use of core values for monitoring hormone levels.

## Conclusion

The use of intraoperative parathyroid hormone monitoring is found to be an effective aid in targeted approaches, resulting in lower morbidity and more successful surgical outcomes. Both collections from peripheral and central sites were found to be effective. The PTH values after the removal of the gland, regardless of the collection site, tend towards homeostasis, showing statistical similarity. However, the reduction in PTH values is different and there is a tendency to use values collected from central sites (Internal Jugular Vein), as they demonstrate greater security for confirming the ideal reduction in values following removal. It is necessary to continue the studies to show the real differences and advantages in relation to the collected sites and their use as standards in PTH drop protocols.

## Funding

None.

## Declaration of competing interest

The authors declare no conflicts of interest.
